# A history of depression and prenatal depression are associated with a lower likelihood of breastfeeding initiation and maintenance, and more breastfeeding problems

**DOI:** 10.1007/s00737-024-01479-5

**Published:** 2024-06-05

**Authors:** Elizabeth C. Braithwaite, Aurora Oftedal, Anne Kaasen, Ziada Ayorech, Mona Bekkhus

**Affiliations:** 1 Department of Psychology, Faculty of Health and Education, Manchester Metropolitan University, Manchester, UK; 2 Department of Children and Families, Division of Mental and Physical Health, Norwegian Institute of Public Health, Oslo, Norway; 3 Faculty of Health Sciences, Oslo Metropolitan University, Oslo, Norway; 4 Promenta Research Centre, Department of Psychology, University of Oslo, Oslo, Norway

**Keywords:** Depression, Breastfeeding initiation, Breastfeeding maintenance, Breastfeeding difficulties, Maternal health

## Abstract

**Purpose:**

This study tests the hypotheses that lifetime history of depression, and prenatal depression, are associated with a reduced likelihood of breastfeeding initiation (giving the baby any breastmilk during the first week of life) and breastfeeding maintenance (giving the baby breastmilk for at least 6 months), and a greater likelihood of reporting breastfeeding problems.

**Methods:**

We analyzed data from the Norwegian Mother, Father, and Child cohort study (MoBa), *N* = 78,307. Mothers reported a lifetime history of depression during the second trimester of pregnancy, and current symptoms of depression during the third trimester using the Hopkins Symptoms Checklist short version (SCL-8). At six months postpartum, mothers self-reported breastfeeding initiation, maintenance, and difficulties.

**Results:**

Using binary logistic regression analyses, we report that a lifetime history of depression is associated with a lower likelihood of breastfeeding initiation (OR = 0.751, 95%CI = 0.650–0.938), breastfeeding maintenance (OR = 0.712, 95%CI = 0.669–0.785), and a greater likelihood of breastfeeding difficulties (OR = 1.86, 95%CI = 1.72–2.06). Similarly, prenatal depression was associated with a lower likelihood of breastfeeding initiation (OR = 0.904, 95%CI = 0.878–0.929), breastfeeding maintenance (OR = 0.929, 95%CI = 0.920–0.938), and a greater likelihood of breastfeeding difficulties (OR = 1.10, 95%CI = 1.09–1.12). Results remained largely unchanged when covaried for several confounding variables, including medication use.

**Conclusion:**

We provide novel evidence that pre-conception and prenatal symptoms of depression are associated with breastfeeding outcomes. This information could be used to identify women very early in pregnancy who may need additional support with breastfeeding. There is also a need to fully understand the biopsychosocial mechanisms that mediate the relationship between depression prior to birth and breastfeeding outcomes.

## Introduction

The World Health Organization (WHO) recommend that infants are exclusively breastfed for the first six months of life because of the many important implications for child and maternal health (Dieterich et al. 2013; WHO 2023). There is a wealth of research that has sought to understand why some women are successful in initiating and maintaining breastfeeding, whereas others are not, so that breastfeeding support can be appropriately targeted (Corby et al. 2021; Jakaitė et al. 2021; Xu et al. 2022). One focused area of research is the reciprocal link between breastfeeding and maternal postnatal mental health. There is good evidence that women who have postnatal depression are less likely to breastfeed (Pope and Mazmanian 2016), and that engagement with breastfeeding may improve mental health symptoms postpartum (Yuen et al. 2022). However, new research suggests that symptoms of depression *preceding* birth may also be associated with breastfeeding practices (Kim et al. 2022), and that antidepressant use during pregnancy is associated with reduced breastfeeding (Gorman et al. 2012; Lewis et al. 2016; Leggett et al. 2017). A recent systematic review and meta-analysis reported that prenatal depression was not associated with breastfeeding initiation, but was associated with a lower likelihood of maintaining breastfeeding for longer than three months (Kim et al. 2022). However, the systematic review was limited by the size and quality of the included studies. Thus, the link between prenatal depression and breastfeeding initiation and maintenance remains unclear. Additionally, no existing research has considered whether a history of depression which *precedes* pregnancy may also be associated with breastfeeding outcomes. If this is the case, healthcare professionals who ask about a lifetime history of poor mental health may be able to identify women who will need extra support with breastfeeding very early in pregnancy.

An additional consideration is *why* women with symptoms of depression, even preceding birth, might be less likely to breastfeed. We know that women with postnatal depression report lower breastfeeding self-efficacy (Dennis 2003; Dennis and McQueen 2007), are more likely to worry about breastfeeding (Chaudron et al. 2001), are more likely to believe that breastfeeding is not the best feeding method for their child (Galler et al. 2006), and are more likely to report breastfeeding difficulties (Tamminen 1988; Jobst et al. 2016; Koutra et al. 2018). Approximately 70% of all mothers’ experience breastfeeding difficulties, such as nipple or breast pain, breast engorgement and mastitis (Gianni et al. 2019). However, it is currently unknown whether prenatal depression and/or depression preceding pregnancy, might be associated with breastfeeding difficulties. If so, this once again highlights an opportunity for early identification of pregnant women who may need additional breastfeeding support.

In the current study, we investigate whether a lifetime history of depression, and/or depression during pregnancy, is associated with breastfeeding initiation, maintenance, and difficulties. Our hypotheses are:

**H1**: A lifetime history of depression will be associated with a lower likelihood of breastfeeding initiation and maintenance, and a greater likelihood of breastfeeding difficulties.

**H2**: Depression during pregnancy will be associated with a lower likelihood of breastfeeding initiation and maintenance, and a greater likelihood of breastfeeding difficulties.

## Method

### Data and participants

The Norwegian Mother, Father, and Child Cohort Study (MoBa) is a population-based pregnancy cohort study conducted by the Norwegian Institute of Public Health. Participants were recruited from all over Norway between 1999 and 2008 (Magnus et al. 2016), of which 41% of pregnant women consented to participate. The cohort includes approximately 114,500 children, 95,200 mothers and 75,200 fathers. The response rate for maternal questionnaires was at 95.1% at 15 weeks gestation, 91.4% at 30 weeks gestation, and 87.0% at 6 months postpartum (Schreuder and Alsaker 2014). This study was based on version 12 of the quality-assured data files released for research in December of 2022. All participants provided informed consent prior to participation. We also used data from The Medical Birth Registry (MBRN), which is a national health registry containing information about all births in Norway (Irgens 2000). The study was approved by the Regional Committees for Medical and Health Research Ethics (REK- 2009/1899-7; 2013/2061; 2020/185,800) and the Norwegian Agency for Shared Services in Education and Research (SIKT).

### Measures

#### Lifetime history of major depression

Lifetime history of major depression was measured during second trimester of pregnancy (week 15) by 6 items that closely correspond to the DSM-III criteria for lifetime major depression (Kendler et al. 1993). Mothers were asked whether they had ever experienced the following for a period of 2 weeks or more (e.g., “felt depressed or sad” or “blamed yourself and felt worthless”) and responded to these by either yes or no. Cronbach’s alpha was 0.85. To meet criteria for lifetime history of depression, participants had to endorse the symptom “felt depressed or sad”, as well as at least two other symptoms, at least three symptoms had to have been present at the same time, and the reason for the symptoms could not be an external event such as a bereavement. Lifetime depression history was coded as a binary variable: 1 (yes) or 0 (no).

#### Prenatal depressive symptoms

Maternal symptoms of depression were assessed at 30 weeks gestation using the SCL-8, a short version of the Hopkins Symptom Checklist (SCL-25) (Tambs and Moum 1993; Tambs and Røysamb 2014). The SCL-8 contains four items assessing anxiety and four items assessing depression. Only the depression subscale was used in the current study. Items such as worrying too much and feeling blue were scored on a Likert scale ranging from 0 (“not bothered”) to 3 (“very bothered”). Cronbach’s alpha for the depression subscale was 0.76.

#### Breastfeeding initiation

Breastfeeding initiation was self-reported at 6 months postpartum. Mothers retrospectively reported how they fed their child during the first week of life. If mothers reported that they had given the child any breastmilk during the first week of life, this was coded as initiating breastfeeding. If mothers reported that they hadn’t given their child any breastmilk during the first week of life, then this was coded as not initiating breastfeeding. Thus, the breastfeeding initiation variable was binary (yes, did initiate vs. no, did not initiate).

#### Breastfeeding maintenance

At six months post-birth, mothers reported what they had given their child to drink or eat during each month of the baby’s life. If mothers who initiated breastfeeding reported that they exclusively or partially gave their child breastmilk up until the sixth month of life, they were coded as maintaining breastfeeding. If mothers who initiated breastfeeding reported that they gave their child breastmilk for up to 5 months or less, this was coded as not maintaining breastfeeding. Thus, the breastfeeding maintenance variable was binary (yes, did maintain vs. no, did not maintain). The 6-month criteria for maintenance of breastfeeding was based on WHO guidelines (WHO 2023).

#### Breastfeeding problems

Mothers reported on breastfeeding problems 6 months after birth. Mothers were asked *“Did you go to your doctor/midwife/health visitor for your own health problems during the first month after the birth?”* and responded yes or no to several health issues including “breastfeeding problems”. Breastfeeding problems were defined as an issue other than sore nipples or mastitis. The breastfeeding problems variable is binary (yes, did experience breastfeeding problems vs no, did not experience breastfeeding problems).

#### Confounders

Medical information including parity, method of delivery (vaginal vs. cesarean birth), gestational age at birth, child’s sex and maternal age at birth were collected using the Medical Birth Registry. Parity was reported as 0 (primiparous), 1, or 2 or more previous births. Whether the woman gave birth by cesarean section was reported yes or no. The length of gestation in days was based on ultrasound estimation, or if ultrasound estimation was not available, gestational length was calculated from the last menstrual period. Child’s sex was coded as male or female. Maternal age at birth was recorded according to the following categories: 17 or younger, 18–19, 20–24, 25–29, 30–34, 35–39, 40–44, or 45 or older. Information about maternal education and use of medication for depression during pregnancy was collected using self-report questionnaires at 15- and 30-weeks gestation. Highest level of completed education was classified according to the following categories: 9-year elementary education, 1–2 years of further education, 3 years of further education, higher education (university or college) up to four years, or higher education more than four years. For use of medication for depression during pregnancy women were asked if they had taken any medication for depression during weeks 0–4, 5–8. 9–12, 13–16, 17–20, 21–24, 25–28, or 29 + of pregnancy. A positive answer at any timepoint was coded as having taken medication for depression during pregnancy.

### Statistical analyses

A series of binary logistic regression equations were calculated to examine the relationship between lifetime history of major depression or prenatal depressive symptoms, and breastfeeding initiation, maintenance, and problems. First, separate analyses examined the predictions of each independent variable on each dependent variable. Second, potential confounders were added to each of the analyses. Confounders included maternal age, gestational age at birth, birth method, child’s sex, maternal education, medication for depression during pregnancy, and parity. Analyses were performed using IBM SPSS version 29 (Statistical Package for the Social Sciences, IBM, Armonk, NY, USA) and R version 4.1.2 (R foundation for Statistical Computing, Vienna, Austria) with the packages “tidyr”, “dplyr”, and “tidyverse”.

## Results

### Sample characteristics

Descriptive statistics for depression, breastfeeding and potential confounders are shown in Table 1. Of 78,307 women who completed the questionnaire at the 6-month postpartum assessment, 76,876 (98.2%) reported that they gave the baby breast milk within the first week of life. Of these mothers, 60,394 (71.3%) reported that they were still giving the baby breast milk at age 6 months, which is in line with national averages (Myhre et al. 2020). A total of 4628 (6%) women reported that they had sought help for breastfeeding problems.

**Table 1:** Descriptive statistics for depression, breastfeeding, and potential confounders

	*N*	%	Mean	SD
**Predictor variables**				
Symptoms of depression in pregnancy	84,853	-	1.25	1.65
Lifetime history of depression	7159	6.5	-	-
**Dependent variables**				
Initiated breastfeeding	76,876	98.2	-	-
Maintained breastfeeding	60,248	78.4	-	-
Sought help for breastfeeding problems	4628	6.0	-	-
**Confounders**				
Maternal age at birth				
24 or younger	8019	10.1	-	-
25–29	25,892	33.1	-	-
30–34	30,607	39.1	-	-
35–39	12,181	15.6	-	-
40 and older	1608	2.1	-	-
Education				
9-year secondary school	1571	2.1	-	-
1–2 years of high school	3234	4.4	-	-
3-year high school	19,626	26.6	-	-
Bachelor’s degree	31,127	42.2	-	-
4 years university or more	18,125	24.6	-	-
Length of gestation (weeks)	77,997	-	39.95	1.73
Caesarean section	10,773	13.8	-	-
Parity				
0 (primiparous)	36,037	46,0	-	-
1	27,583	35,2	-	-
2 or more	14,687	18,8	-	-
Medication for depression in pregnancy	792	1.0		

### Breastfeeding initiation

Higher symptoms of prenatal depression were associated with a lower likelihood of initiating breastfeeding during the first week of the baby’s life, see Table 2. Results remained unchanged after adjusting for confounders. Lifetime history of depression was also associated with a lower likelihood of initiating breastfeeding, but this association became non-significant when confounders were added to the model. Of the potential cofounders, a longer gestational period, higher education, having previous children, and having a female baby were associated with a greater likelihood of breastfeeding initiation, while older maternal age, having a cesarean section, and using medication for depression during pregnancy were associated with a lower likelihood of breastfeeding initiation.

**Table 2:** Summary of binary logistic regression models of depression in pregnancy and lifetime history of depression in predicting breastfeeding initiation

	Log-odds coefficient and 95% CI	*p*-value
**Breastfeeding initiation** (*n* = 78,307)		
*Without confounders*		
Depression in pregnancy	0.904 (0.878 − 0.929)	< 0.001
Constant	63.72	< 0.001
Lifetime history of depression	0.781 (0.650 − 0.938)	0.008
Constant	54.8	< 0.001
*With confounders*		
Parity	1.14 (1.06–1.23)	< 0.001
Caesarian section	0.784 (0.695 − 0.806)	< 0.001
Gestational length	1.03 (1.02–1.03)	< 0.001
Female child	1.16 (1.03–1.30)	0.011
Maternal age	0.880 (0.825 – 0.939)	< 0.001
Education	1.29 (1.23–1.34)	< 0.001
Medication for depression	0.523 (0.359 − 0.763)	< 0.001
Depression 30 weeks	0.932 (0.905 − 0.961)	< 0.001
Constant	0.030	< 0.001
Parity	1.14 (1.06–1.22)	< 0.001
Caesarian section	0.744 (0.692 − 0.799)	< 0.001
Gestational length	1.02 (1.02–1.03)	< 0.001
Female child	1.16 (1.04–1.29)	0.009
Maternal age	0.880 (0.827 − 0.936)	< 0.001
Education	1.32 (1.26–1.37)	< 0.001
Medication for depression	0.452 (0.316 − 0.645)	< 0.001
Lifetime history of depression	0.912 (0.750–1.11)	0.356
Constant	0.035	< 0.001

### Breastfeeding maintenance

Both prenatal depression and lifetime history of depression predicted a lower likelihood of maintaining breastfeeding at age 6 months, and results were unchanged following addition of confounders, see Table 3. Older maternal age, longer gestational period, having previous children, higher maternal education and having a female baby were associated with a greater likelihood of breastfeeding maintenance, whereas giving birth by caesarian section was associated with a lower likelihood of breastfeeding maintenance.

**Table 3:** Summary of binary logistic regression models of depression in pregnancy and lifetime history of depression in predicting breastfeeding maintenance

	Log-odds coefficient and 95% CI	*p*-value
**Breastfeeding initiation** (*n* = 76,876)		
*Without confounders*		
Depression in pregnancy	0.929 (0.920 − 0.938)	< 0.001
Constant	3.79	< 0.001
Lifetime history of depression	0.712 (0.669 − 0.785)	< 0.001
Constant	3.39	< 0.001
*With confounders*		
Parity	1.15 (1.12–1.17)	< 0.001
Caesarian section	0.784 (0.750 − 0.820)	< 0.001
Gestational length	1.01 (1.00–1.01)	< 0.001
Female child	1.07 (1.03–1.11)	0.011
Maternal age	1.10 (1.07–1.12)	< 0.001
Education	1.47 (1.45–1.49)	< 0.001
Medication for depression	0.867 (0.727–1.03)	0.112
Depression 30 weeks	0.963 (0.952 − 0.974)	< 0.001
Constant	0.087	< 0.001
Parity	1.15 (1.12–1.17)	< 0.001
Caesarian section	0.790 (0.757 − 0.825)	< 0.001
Gestational length	1.01 (1.00–1.01)	< 0.001
Female child	1.07 (1.03–1.11)	< 0.001
Maternal age	0.880 (0.827 − 0.936)	< 0.001
Education	1.47 (1.45–1.50)	< 0.001
Medication for depression	0.844 (0.712–1.00)	0.051
Lifetime history of depression	0.801 (0.750 – 0.857)	< 0.001
Constant	0.078	< 0.001

### Breastfeeding problems

Women with prenatal depression, and women with a lifetime history of depression, were more likely to have asked for help with breastfeeding problems than women with no history of depression or without prenatal depression, see Table 4. Results remained unchanged after controlling for confounders. Women who were older, more educated and who had a longer gestational period were more likely to report having had problems, while higher parity was associated with fewer breastfeeding problems. Mothers who initiated breastfeeding within a week following the birth, but who did not maintain breastfeeding to six months post-birth, were more likely to have sought help for breastfeeding problems than mothers who did maintain breastfeeding to six months, X^2^(1) = 553.4, *p* < .001.

**Table 4:** Summary of binary logistic regression models of depression in pregnancy and lifetime history of depression in predicting breastfeeding problems

	Log-odds coefficient and 95% CI	*p*-value
**Breastfeeding initiation** (*n* = 76,876)		
*Without confounders*		
Depression in pregnancy	1.10 (1.09–1.12)	< 0.001
Constant	0.056	< 0.001
Lifetime history of depression	1.88 (1.72–2.06)	< 0.001
Constant	0.06	< 0.001
*With confounders*		
Parity	0.458 (0.434 − 0.483)	< 0.001
Caesarian section	0.993 (0.918 − 1.08)	0.866
Gestational length	1.01 (1.00–1.01)	< 0.001
Female child	0.968 (0.908–1.03)	0.323
Maternal age	1.10 (1.06–1.15)	< 0.001
Education	1.10 (1.07–1.13)	< 0.001
Medication for depression	0.947 (0.704–1.27)	0.719
Depression 30 weeks	1.13 (1.11–1.15)	< 0.001
Constant	0.007	< 0.001
Parity	0.475 (0.451 − 0.500)	< 0.001
Caesarian section	0.994 (0.920–1.07)	0.869
Gestational length	1.01 (1.00–1.01)	< 0.001
Female child	0.969 (0.911–1.03)	0.324
Maternal age	1.09 (1.05–1.13)	< 0.001
Education	1.09 (1.06–1.12)	< 0.001
Medication for depression	1.09 (0.830–1.43)	0.532
Lifetime history of depression	1.95 (1.76–2.15)	< 0.001
Constant	0.011	< 0.001

## Discussion

We tested the hypotheses that a lifetime history of depression, and symptoms of depression during pregnancy, would be associated with a reduced likelihood of breastfeeding initiation and maintenance, and a greater likelihood of experiencing breastfeeding problems. Our hypotheses were mainly supported. Prenatal depression was associated with a lower likelihood of breastfeeding initiation. Of the women who did initiate breastfeeding, those with a lifetime history of depression and those with prenatal depression were less likely to maintain breastfeeding to 6 months and were more likely to self-report breastfeeding problems. These associations remained significant when controlling for prenatal antidepressant use, which has previously been reported to be associated with reduced breastfeeding initiation (Grzeskowiak et al. 2022). However, just 1% of the sample reported using antidepressant use during pregnancy, therefore it is not completely clear how medication use might impact breastfeeding given the confounding of maternal mood.

Our results are partly in line with a recent systematic review and meta-analysis which reported that prenatal depression was associated with a reduced likelihood of breastfeeding maintenance past 3 months, but not with breastfeeding initiation (Kim et al. 2022). The meta-analysis defined breastfeeding initiation as giving the child breastmilk on the first day following birth, whereas we considered breastfeeding initiation to be any breastmilk given in the first week post-birth. Thus, it is possible that an increased variability in our data, from assessing breastfeeding initiation over a longer period, allowed us to detect this effect at a level of accepted statistical significance, though the odds ratio reported in the meta-analysis was similar to what we report in this study (0.7). In line with the meta-analysis, we also report that prenatal depression was associated with a lowered likelihood of breastfeeding maintenance to 6 months post birth.

To our knowledge, this is the first study to show that a lifetime history of depression is associated with breastfeeding maintenance. It may therefore be possible to identify women very early in pregnancy who may need additional breastfeeding support, simply by asking about depression history. We also report, for the first time, that a lifetime history of depression, and prenatal depression, are associated with more breastfeeding difficulties. Reports of breastfeeding difficulties in westernized populations are as high as 70% (Gianni et al. 2019). We know that many women feel pressured to breastfeed (Lagan et al. 2014), and in Norway specifically, there are large cultural values around breastfeeding which could also contribute to negative feelings when a mother does not breastfeed (Hvatum and Glavin 2017). Often judgement, stigma, distress and guilt are experienced by mothers who do not breastfeed (Jackson et al. 2022), and it has been hypothesized that this might contribute to the onset of new, or worsening of existing, mental health problems (Gianni et al. 2019).

One possible explanation as to why a history of depression and prenatal depression are associated with more breastfeeding difficulties is that women who experience depression also have physiological changes that make it more difficult to breastfeed. We know that women who have postnatal depression have impaired milk production and ejection from the nipple (Ziomkiewicz et al. 2021), changes in breastmilk composition (Ziomkiewicz et al. 2021) and produce a lower total milk volume (Syam et al. 2021). We also know that several hormones are associated with the stimulation of milk ejection and breastmilk volume, especially oxytocin (Uvnäs Moberg et al. 2020). The gene that codes for the oxytocin receptor (OXTR) is susceptible to stress-related epigenetic regulation of gene expression (Kumsta et al. 2013; Kraaijenvanger et al. 2019), and that mothers with perinatal depression have elevated DNA methylation at OXTR compared with mothers with no perinatal depression (King et al. 2017). It is therefore reasonable to hypothesize that depression prior to birth could result in the alteration of DNA methylation at genes which are important for breastfeeding, such as OXTR, which in turn is associated with poor milk ejection and reduced milk volume. This could explain why women with pre-conception depression are less likely to initiate and maintain breastfeeding, and also experience more problems. This is a novel and important avenue of future research in this field. It is also important to consider that breastfeeding (Colodro-Conde et al. 2015), depression (Sullivan et al. 2000), and many of our potential confounders, for example educational attainment (Okbay, Wu et al. 2022), are themselves under substantial genetic influence and so it is possible that there is overlap in the genes influencing both the exposure and outcome, and this genetic confounding is partially driving the positive associations reported here.

Strengths of this study include the very large sample size which is powered to detect small effects. Breastfeeding outcomes in this cohort are very similar to the Norwegian national average (98% initiation and 78% maintenance (Myhre et al. 2020), which gives confidence in the generalizability of our reported findings. However, a limitation of collecting data from large cohorts is that often measures are brief and almost always rely on self-report. The measures of maternal mental health and breastfeeding used in this study were all based on self-report and therefore subject to reporter bias and recall bias. The assessment of breastfeeding difficulties was based on just one item, and therefore more research is needed to understand some of the more contextual complexities around breastfeeding difficulties. Most of our knowledge about the relationship between breastfeeding and maternal mental health is based on postnatal depression. An important consideration is that lifetime history of depression and prenatal depression are both highly correlated with postnatal depression. For this reason, we did not control for postnatal depression in main analyses, to avoid multicollinearity. We did however conduct a sensitivity analysis that included maternal postnatal depression (assessed at 6 months postpartum) as a confounder in the regression models, and the findings remained largely unchanged (not presented here).

To conclude, we provide novel evidence from a large population study that a lifetime history of depression, and prenatal depression, is associated with a lower likelihood of breastfeeding initiation and maintenance, and more breastfeeding difficulties. This knowledge could be used to identify women very early during pregnancy for targeted breastfeeding support strategies. An important area for future scholarship is furthering our understanding of the biopsychosocial mechanisms by which depression prior to birth might be related to early breastfeeding cessation and more breastfeeding problems.

## Data Availability

Data from the Norwegian Mother, Father and Child Cohort Study and the Medical Birth Registry of Norway used in this study are managed by the national health register holders in Norway (Norwegian Institute of public health) and can be made available to researchers, provided approval from the Regional Committees for Medical and Health Research Ethics (REC), compliance with the EU General Data Protection Regulation (GDPR) and approval from the data owners. The consent given by the participants does not open for storage of data on an individual level in repositories or journals. Researchers who want access to data sets for replication should apply through helsedata.no. Access to data sets requires approval from The Regional Committee for Medical and Health Research Ethics in Norway and an agreement with MoBa.
